# Activation of Host Translational Control Pathways by a Viral Developmental Switch

**DOI:** 10.1371/journal.ppat.1000334

**Published:** 2009-03-20

**Authors:** Carolina Arias, Derek Walsh, Jack Harbell, Angus C. Wilson, Ian Mohr

**Affiliations:** 1 Department of Microbiology and NYU Cancer Institute, New York University School of Medicine, New York, New York, United States of America; 2 National Institute For Cellular Biotechnology, Dublin City University, Dublin, Ireland; University of Wisconsin-Madison, United States of America

## Abstract

In response to numerous signals, latent herpesvirus genomes abruptly switch their developmental program, aborting stable host–cell colonization in favor of productive viral replication that ultimately destroys the cell. To achieve a rapid gene expression transition, newly minted capped, polyadenylated viral mRNAs must engage and reprogram the cellular translational apparatus. While transcriptional responses of viral genomes undergoing lytic reactivation have been amply documented, roles for cellular translational control pathways in enabling the latent-lytic switch have not been described. Using PEL-derived B-cells naturally infected with KSHV as a model, we define efficient reactivation conditions and demonstrate that reactivation substantially changes the protein synthesis profile. New polypeptide synthesis correlates with 4E-BP1 translational repressor inactivation, nuclear PABP accumulation, eIF4F assembly, and phosphorylation of the cap-binding protein eIF4E by Mnk1. Significantly, inhibiting Mnk1 reduces accumulation of the critical viral transactivator RTA through a post-transcriptional mechanism, limiting downstream lytic protein production, and impairs reactivation efficiency. Thus, herpesvirus reactivation from latency activates the host cap-dependent translation machinery, illustrating the importance of translational regulation in implementing new developmental instructions that drastically alter cell fate.

## Author Summary

Kaposi's sarcoma-associated herpesvirus (KSHV) is an important human pathogen and, like all herpesviruses, establishes a state of permanent residency in the infected host called latency. Major sites of KSHV latency are cells of the immune system and cells lining blood vessels. In individuals with weakened immunity, inappropriate growth of these cells driven by the resident virus can give rise to primary effusion lymphoma and Kaposi's sarcoma, respectively. These life-threatening cancers are most common in patients with HIV/AIDS and have become a major source of mortality in parts of sub-Saharan Africa. Under appropriate stimuli, herpesviruses change their relationship with the host cell and begin to manufacture proteins required to assemble new infectious virus particles that can be released and spread. To achieve this, the virus hijacks key processes within the cell and conscripts them into producing viral proteins. In this study, we describe for the first time how KSHV carefully manipulates the host protein synthesis machinery during the switch from latency to this specialized infectious virus production mode. Our results show that although overall protein synthesis is diminished, key components of the host's protein manufacturing machinery are actually stimulated, presumably to accelerate viral protein production.

## Introduction

Regulation of gene expression at the level of mRNA translation is important for the control of numerous biological processes including cell growth, differentiation, development and the response to environmental stress [1],[2]. Unlike prokaryotes, the vast majority of eukaryotic mRNAs are unable to recognize ribosomes directly and rely instead on an intricate set of translation initiation factors that assemble a specialized multisubunit complex onto the mRNA 5′ terminus to recruit the 40S ribosome subunit. For 7-methyl GTP capped mRNAs, this involves eIF4F, a tripartite complex that includes a cap-binding subunit (eIF4E) and an RNA helicase (eIF4A) tethered to eIF4G, a large molecular platform that contacts the eIF3-bound 40S subunit. In addition, eIF4G associates with other translational control proteins such as the eIF4E kinase Mnk1 and the poly(A)-binding protein (PABP), which directly binds the mRNA 3′ end and indirectly contacts the 5′ end through eIF4G. Moreover, the responsiveness of individual constituents of this complex to a wide spectrum of cellular signals allows the translational machinery to respond rapidly to diverse physiological effectors [3]. The 4E-BP translational repressor family, for example, sequesters eIF4E and prevents binding to eIF4G, limiting ribosome recruitment. Similarly, the ERK and p38-responsive eIF4G-associated kinase Mnk1 modulates eIF4E phosphorylation, which in specific instances has been associated with increased translation rates [4]–[7]. Thus, regulated translation initiation factor complex assembly and modification is poised to potentiate important developmental decisions by controlling global and specific mRNA translation.

Viruses provide attractive models to study simple developmental decisions. In prokaryotes, much has been learned using bacteriophage λ to investigate how the lysis-lysogeny decision is made [8]. In eukaryotes, latent herpesviruses exist in one of two developmental states within their hosts and must resolve an analogous question of whether to remain latent or initiate productive viral growth. Different herpesviruses permanently colonize distinct specialized host cell-types. Those that establish residency in dividing cell populations, exemplified by members of the γ-herpesvirus subfamily that includes Kaposi's sarcoma associated herpesvirus (KSHV/HHV8), express a limited subset of viral genes that stimulate cell proliferation, allow for viral minichromosome replication and segregation, and evade antiviral defenses [9]. In response to poorly understood environmental cues, these viruses can switch to a developmental program that results in productive replication. This alternate pathway involves activating a temporally coordinated cascade of viral lytic gene expression, which in turn results in massive viral DNA amplification, progeny virus production, and ultimately host cell destruction [10],[11]. To effectively switch its gene expression program, all herpesviruses produce a new population of viral mRNAs transcribed by the cellular RNA polymerase II, which are mostly capped and polyadenylated like their host counterparts [12]–[16], and these must successfully engage and reprogram the host cell translational apparatus. This is a critical component of the developmental switch because viruses are absolutely dependent upon the translational machinery resident in their hosts [17]. Manipulating host translation initiation factors to ensure that nascent viral mRNAs successfully recruit ribosomes will therefore determine the overall level and efficacy with which the newly transcribed developmental instructions are executed. While we have a general understanding of how the viral transcriptome is altered for many viruses, a role for translational control in the developmental switch from a latent to a productively replicating state has not been described.

One of the earliest events in the genetic switch that initiates productive KSHV replication is the accumulation of RTA, a transcription factor encoded by ORF50 [18]–[20]. By recruitment to promoter elements in the viral genome, RTA triggers a complex program of viral mRNA synthesis resulting in replication and assembly of infectious viral particles. Here we establish efficient conditions for KSHV reactivation that reveal major alterations in the protein synthesis profile of a naturally infected B-cell line. These changes are accompanied by inactivation of the 4E-BP1 translational repressor, eIF4F assembly, and eIF4E phosphorylation. Moreover, inhibiting the eIF4E kinase Mnk1 impaired viral entry into the lytic cycle and reduced the overall abundance of the master viral activator RTA via a post-transcriptional mechanism. Together, these results establish a role for remodeling and modifying cellular translation initiation factor complexes in orchestrating global changes in protein synthesis during reactivation of latent viruses, and highlight the importance of translational as well as transcriptional control in executing a simple developmental decision.

## Results

### Efficient reactivation of KSHV in response to combined stimuli in engineered cell lines

Much of our knowledge of the developmental switch that maintains latency or promotes productive viral replication has been garnered from studies of primary effusion lymphoma (PEL)-derived B-cell lines (such as BCBL1 cells) latently-infected with KSHV. While KSHV lytic replication can be induced by ectopic expression of the viral transactivator RTA or treatment with chemicals such as phorbol esters (TPA) or sodium butyrate, the low efficiency with which reactivation is induced severely limits the system's utility because only a small number of cells undergo lytic replication at any given moment. In hopes of obtaining greater levels of reactivation from latency, we turned to an inducible BCBL1-derived cell line (TREx BCBL1-RTA) developed by Jung and colleagues that expresses epitope-tagged RTA under the control of a tetracycline-inducible promoter [21]. One limitation of this system is that it cannot address the contribution, if any, of early nuclear events in RTA mRNA biogenesis that precede cytoplasmic mRNA accumulation such as transcription template effects, differential mRNA splicing, and nuclear export. Through a well-described positive feedback loop, expression of RTA from the cDNA incorporated into the cellular genome also induces the viral genomic ORF50 locus and therefore a mixture of ectopic and natural RTA transcripts are produced in the reactivating cells [19],[21],[22].

**Figure 1:**
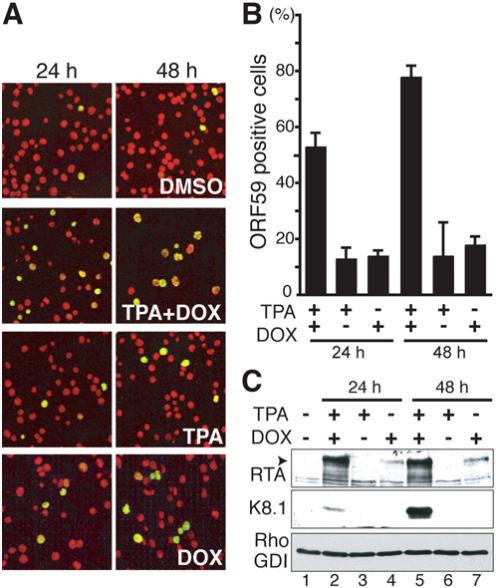
Efficient KSHV reactivation from naturally infected PEL-derived TREx BCBL1-RTA cells. A. Immunofluorescence analysis of KSHV lytic reactivation. PEL-derived TREx BCBL1-RTA cells were treated with TPA, doxycycline (DOX) or TPA+DOX for 24 h or 48 h. Fixed, permeabilized cells were then immunostained with antisera specific for the KSHV delayed-early protein ORF59 (green signal), a marker of KSHV lytic replication, and the DNA counterstained with propidium iodide (red signal). The photographs show representative fields of independent, triplicate experiments. B. The percentage of ORF59-positive cells was quantified by counting at least 1000 cells from the samples in A. Error bars: Standard deviations from independent, triplicate experiments. C. Accumulation of KSHV lytic gene products in TREx BCBL1-RTA cells induced to reactivate. Total protein isolated from the samples described in A and B was fractionated by SDS-PAGE and analyzed by immunoblotting using the indicated antisera. The arrowhead indicates the mobility of full-length RTA. Cellular RhoGDI serves as a loading control.

**Figure 2:**
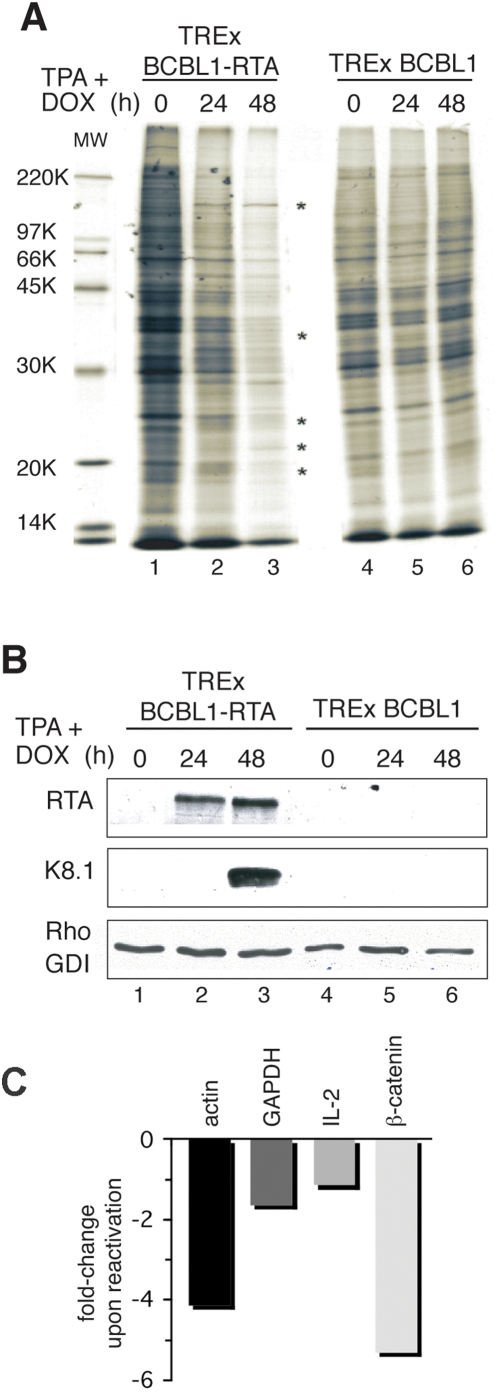
KSHV lytic reactivation changes the ongoing protein synthesis profile in naturally infected, PEL-derived B-cells. A. TREx BCBL1 and TREx BCBL1-RTA cells were treated with TPA+DOX and metabolically labeled for 30 min with ^35^S amino acids at the indicated times post-induction. Total protein was isolated, fractionated by SDS-PAGE and the fixed, dried gel exposed to X-ray film. Asterisks (*) indicate newly synthesized proteins detected only in TREx BCBL1-RTA cells induced with TPA+DOX. Molecular weight standards appear in the leftmost lane and their sizes (in KDa) are indicated in the margin. B. Same as in A except the samples were analyzed by immunoblotting using the indicated antisera. Cellular RhoGDI serves as a loading control. C. Total RNA was isolated from TREx BCBL-1-RTA cells treated with DMSO or induced with TPA+DOX for 48 h. Changes in the overall relative abundance of the indicated representative cellular mRNAs in uninduced (DMSO-treated) vs TPA+DOX-induced cells was measured by real-time RT-PCR analysis.

To evaluate reactivation in TREx BCBL1-RTA cells in response to TPA, or the tetracycline analog doxycycline (DOX), we scored ORF59 delayed-early lytic gene product accumulation by immunofluorescence. The overwhelming majority of the initial starting cultures were latently infected with KSHV, as all the cells were positive for LANA, a KSHV latent infection marker (not shown), and less than 5% exhibited staining for ORF59 (Fig. 1A). This basal ORF59 staining reflects the low spontaneous lytic reactivation routinely seen in these cultures. While ORF59 accumulated between 24–48 h post-induction with TPA or DOX alone, this was limited to only 15–20% of the cells (Fig. 1A, B) and was similar to the level observed in the parental TREx BCBL-1 line treated with TPA alone. Combining both stimuli (TPA+DOX) resulted in a synergistic response where more than 50% of the TREx BCBL1-RTA population was ORF59 positive at 24 h post-induction, and this approached 80% of the cells after 48 h (Fig. 1 A, B). Inducible reactivation of this magnitude in a naturally infected, KSHV-positive PEL B-cell line has not been reported previously. The accumulation of other viral proteins, notably the critical transactivator RTA and the late glycoprotein K8.1 paralleled the ORF59 increase (Fig. 1C, lanes 2,5). Significantly, whereas K8.1 levels after 48 h TPA+DOX treatment were robust, the protein was not detected in cultures treated only with TPA or DOX individually (Fig. 1C, lanes 3–4, 6–7). Expression of the K8.1 late gene is dependent upon prior DNA synthesis and is thus a “true-late” lytic marker that reflects the progression of the viral lytic cycle well beyond the point of ORF59 expression. Furthermore, accumulation of slower migrating, glycosylated forms of K8.1 from 48 h onward (e.g., see panel B of figure four), indicative of their maturation as they traffic through the cellular secretory pathway, is consistent with progression of the lytic program into later phases of the productive growth cycle. Lastly, infectious virus production was confirmed by applying cell-free lysates prepared from TREx-BCBL1-RTA cells either treated with DMSO or induced with TPA+DOX onto a 293 PAN-luc reporter cell line which expresses the firefly luciferase gene under the control of the RTA-responsive PAN promoter [23]. Activation of an RTA-responsive gene in a reporter cell line following application of cell-free supernatants is a powerful indicator of infectious virus production [23],[24]. Luciferase activity was exclusively detected in lysates prepared from TPA+DOX-treated cultures, establishing that only lysates from TPA+DOX treated cultures were capable of activating an RTA-responsive reporter gene, and in turn suggests that RTA-delivery was mediated by infectious KSHV particles in the cell-free lysate (Fig. S1). Thus, the reactivation conditions used throughout this study do result in infectious virus production, which correlates well with ORF59 and K8.1 accumulation, our two other independent experimental markers for reactivation. Lytic reactivation in TREx BCBL1-RTA cultures treated individually with DOX or TPA was not simply delayed compared to treatment with both drugs because the number of ORF59-positive cells only increased beyond the 20% mark after prolonged incubation and was accompanied by massive cell death, hindering further analysis (not shown). As a control, viral reactivation was significantly lower after TPA+DOX treatment of the parental cell line TREx BCBL1, which does not express ectopic RTA (not shown; Fig. 2B). Thus, TREX BCBL1-RTA cultures treated with both TPA+DOX induced a much larger fraction of cells to enter the productive growth cycle as measured by early and late viral lytic gene expression.

### Reactivation from latency alters the protein synthesis profile in naturally infected PEL-derived B-cells

**Figure 3:**
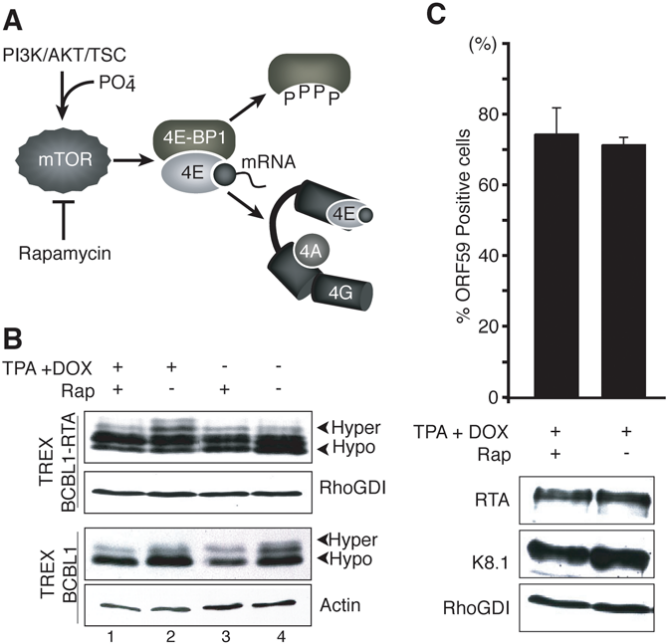
Phosphorylation of the translational repressor 4E-BP1 in response to KSHV lytic reactivation. A. Activation of the PI3-Kinase (PI3K) /AKT/TSC pathway by diverse stimuli results in phosphorylation and subsequent inactivation of 4E-BP1 by mTORC1. Hyperphosphorylated 4E-BP1 releases the cap-binding protein eIF4E (shown bound to the mRNA 5′ cap), making it available to bind eIF4G and assemble a functional eIF4F complex composed of eIF4E, eIF4G, and eIF4A. The macrolide rapamycin inhibits mTORC1 and prevents 4E-BP1 phosphorylation. B. Hyperphosphorylated 4E-BP1 accumulates following KSHV reactivation. TREx BCBL1 or TREx BCBL1-RTA cells were untreated (−) or treated (+) with TPA+DOX in the presence or absence of rapamycin (Rap). At 48 h post-induction, total protein was harvested, fractioned by SDS-PAGE in a 17.5% gel and analyzed by immunoblotting using anti-4E-BP1. Arrowheads indicate the differential electrophoretic migration of the hypo- and hyper-phosphorylated 4E-BP1 forms. C. Inhibition of 4E-BP1 phosphorylation does not affect KSHV reactivation. Top panel: TREx BCBL-1 cells were induced with TPA+DOX in the presence and absence of rapamycin (rap). The fraction of ORF59 positive cells was quantified as described in the legend to Fig. 1. Bottom panel: Total protein was isolated, fractionated by SDS-PAGE and analyzed by immunoblotting using the indicated antisera. RhoGDI: loading control.

Having established conditions where 80% of the latently-infected cells in the population were routinely induced to enter the KSHV lytic cycle, it was now possible to directly evaluate how switching from latency to lytic replication altered the profile of polypeptides produced in a naturally infected PEL-derived cell line. To characterize cellular protein synthesis following KSHV reactivation from latency in the PEL-derived B-cells, TREx BCBL1 and TREx BCBL1-RTA cells were treated with a combination of TPA+DOX for 24 or 48 h. After metabolic labeling with ^35^S amino acids for 30 min., total protein was isolated, fractionated by SDS-PAGE, and visualized by autoradiography. By 24 h post-induction, a modest reduction in ^35^S-incorporation into newly synthesized proteins was evident in TREx BCBL1-RTA cells (Fig. 2A). While numerous bands were reduced in intensity compared to the 0 h point, new polypeptide species migrating at different positions relative to the molecular weight standards were clearly visible (Fig. 2A). Some of these proteins continued to be produced at a similar rate despite the continued overall suppression of protein synthesis evident at 48 h post-induction. The selective, sustained accumulation of these new polypeptide species while global mRNA translation rates are suppressed suggests that they represent de novo synthesis of viral factors or cellular proteins produced in response to active infection (Fig. 2A). Further investigation will be necessary to determine the precise identity and nature of these proteins. The global suppression of host protein synthesis triggered by lytic reactivation, however, could involve accelerated mRNA decay, as shown for the KSHV ORF37 (SOX) gene product in immortalized endothelial cells [25]. Significantly, the relative abundance of a representative set of cellular mRNAs (β-actin, GAPDH, IL-2, β-catenin) measured by real-time PCR after 48 h of TPA+DOX treatment was only modestly reduced between one to five-fold compared with control, uninduced samples (Fig. 2C). Whilst these changes in mRNA steady-state levels will most likely contribute to the observed impairment of global mRNA translation upon KSHV lytic reactivation, the comparatively modest extent to which mRNA levels decline may not be sufficient to fully account for the reduced translation observed in Fig. 3A. In contrast, protein synthesis proceeded unaltered in the TPA+DOX-treated parental TREx BCBL1 cells (Fig. 2A) or TREx BCBL1-RTA cells individually treated with DOX alone (not shown). Finally, the accumulation of specific viral proteins, including K8.1, was only observed in TREx BCBL1-RTA cells, but not in the parental TREx BCBL1 cells, in response to combined TPA+DOX treatment (Fig. 2B). Together, these observations suggest that the switch of KSHV from one developmental state (latency) to an alternative state (lytic replication) is accompanied by a strong suppression of host protein synthesis. This represents the first demonstration that inducting lytic replication in a naturally infected PEL-derived cell line suppresses overall protein synthesis.

### Inactivation of the 4E-BP1 translational repressor during reactivation from latency

Given the significant changes to global protein synthesis rates following reactivation from latency, we investigated if modification of the cellular cap-dependent translational machinery was associated with and contributes to reactivation. To this end, we first focused on 4E-BP1, a translational repressor that regulates the availability of the cap-binding protein eIF4E [3]. In its hypophosphorylated form, 4E-BP1 sequesters eIF4E and thereby prevents eIF4G binding, eIF4F complex assembly, and 40S subunit recruitment without affecting eIF4E binding to the 5′ cap structure. 4E-BP1 phosphorylation by mTORC1 liberates eIF4E, allowing it to bind eIF4G and assemble a functional eIF4F complex, which in turn mediates 40S ribosome subunit recruitment to the capped 5′ mRNA terminus (Fig. 3A). To determine how KSHV reactivation affects 4E-BP1 phosphorylation, total protein isolated from TPA+DOX-treated TREx BCBL1-RTA cells or the parental TREx BCBL1 line was fractionated by SDS-PAGE in a 17.5% gel capable of resolving 4E-BP1 phosphorylated isoforms and visualized by immunoblotting using anti-4E-BP1 antibodies. After 48 h of treatment, slower migrating bands corresponding to hyperphosphorylated 4E-BP1 were observed (Fig. 3B, compare lanes 2 and 4). Moreover, hyperphosphorylated 4E-BP1 accumulation was not detected in the TPA+DOX-treated parental TREx BCBL1 cells, suggesting that 4E-BP1 hyperphosphorylation is dependent on viral reactivation and not on the pharmacological treatment of the cells per se. Rapamycin, an inhibitor of the cellular kinase mTORC1, prevented hyperphosphorylated 4E-BP1 accumulation demonstrating a requirement for mTORC1 activity (Fig. 3B). mTORC1 stimulation could result from viral and/or cellular effectors acting upstream of or directly on mTORC1 components. The preponderance of hypophosphorylated 4E-BP1 in TREx BCBL1 and uninduced TREx BCBL1-RTA cells, irrespective of rapamycin treatment indicates that basal mTORC1 activity in these latently infected, actively dividing, transformed PEL cells is limited (Fig. 3B). However, despite efficiently reducing 4E-BP1 phosphorylation, preventing inactivation of the repressor 4E-BP1 with rapamycin did not significantly change the fraction of TREx BCBL1-RTA cells capable of supporting KSHV lytic reactivation, as determined by ORF59 immunofluorescence and immunoblotting for RTA or K8.1 (Fig. 3C). This suggests that the overall levels of the 4E-BP1 repressor may not be capable of sequestering enough eIF4E to have a detectable effect in these transformed PEL cell lines. Nevertheless, the induction of the viral lytic program is capable of inactivating the 4E-BP1 translational repressor.

### Reactivation from latency promotes eIF4F assembly

**Figure 4:**
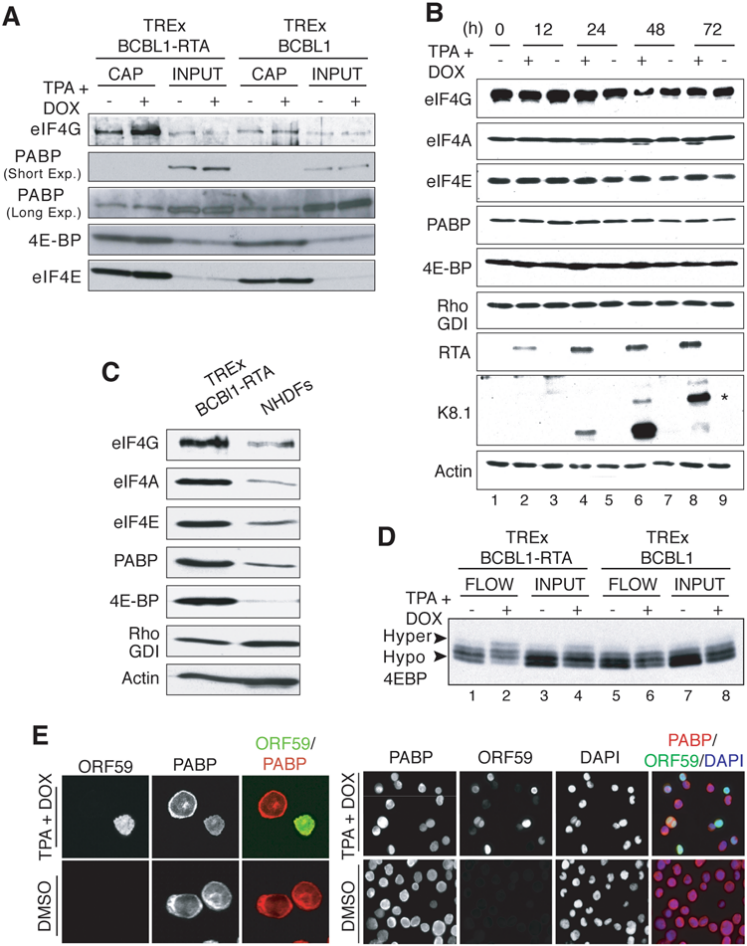
Lytic reactivation of KSHV promotes eIF4F assembly. A. KSHV lytic reactivation increases the incorporation of eIF4E into the eIF4F complex. Non-ionic detergent lysates (INPUT) prepared from TREx BCBL1 or TREx BCBL1-RTA cells either untreated (−) or treated (+) with TPA+DOX for 48 h prior to harvesting were incubated with 7-methyl-GTP (7-M GTP) sepharose. The 7-M GTP cap-bound proteins (CAP) were fractionated by SDS-PAGE and analyzed by immunoblotting using the indicated antisera. B. The abundance of eIF4F-core and associated components remains constant in TREx BCBL1-RTA cells induced to reactivate. Total protein isolated from TREx BCBL1-RTA cells untreated (−) or treated (+) with TPA+DOX for the indicated times was fractionated by SDS-PAGE and analyzed by immunoblotting for the indicated proteins. The asterisk indicates the slower-migrating fully-glycosylated, mature form of K8.1. RhoGDI and actin: loading controls. C. TREx BCBL1-RTA cells contain relatively high levels of eIF4F core and associated proteins. Total protein isolated from equal numbers of live, untreated TREx BCBL1-RTA or primary NHDF cells (1.5×10^6^/ml) was fractionated by SDS-PAGE and analyzed by immunoblotting using the indicated antisera. D. Enrichment of 4E-BP1 hyperphosphorylated isoforms in the 7-M GTP sepharose unbound fraction upon lytic reactivation. As in A except the 7-M GTP sepharose unbound, flow-through fraction (FLOW) was fractionated by SDS-PAGE in 17.5% gels to resolve phosphorylated 4E-BP1 isoforms (hyper- *vs.* hypophosphorylated indicated by arrowheads) and analyzed by immunoblotting using anti-4E-BP1 antisera. E. PABP redistribution during KSHV lytic reactivation. Left panel: At 48 h post-induction, TREx BCBL1-RTA cells treated as described in A were collected, and immunostained using antisera specific for ORF59 (green signal) or PABP (red signal). Images were viewed with Zeiss LSM510 Meta confocal microscope, and colocalization was evaluated. Right panel: Cells were processed and immunostained for ORF59 and PABP as indicated for the left panel, but additionally counterstained with DAPI and viewed with a Zeiss Axiovert fluorescence microscope. For better contrast, single-stain images are shown in black & white. The Rightmost panel is a merged image showing ORF59 (green), PABP (red), and DAPI (blue).

While inactivation of the repressor 4E-BP1 can modulate eIF4E availability, its ability to regulate translation depends upon its overall abundance. In addition, repressor inactivation is not always accompanied by eIF4F assembly, which requires the association of eIF4E with eIF4G [26]. To determine if KSHV reactivation can influence eIF4F complex formation, eIF4E-associated proteins present in non-ionic detergent lysates prepared from TPA+DOX-treated TREx BCBL1 and TREx BCBL1-RTA cells were isolated by batch chromatography on 7-M GTP sepharose. After washing, bound polypeptides were fractionated by SDS-PAGE and analyzed by immunoblotting. Remarkably, the overall abundance of eIF4G retained (by eIF4E) on the 7-M GTP matrix increased in TREx BCBL1-RTA cells induced to reactivate with TPA+DOX, whereas changes in the amount of the cap-binding protein eIF4E or PABP in the bound fraction were not observed (Fig. 4A). No differences in the amounts of eIF4E, eIF4G, or PABP bound to eIF4E were detected in lysates prepared from TPA+DOX-treated parental TREx BCBL1 cells (Fig. 4A). Taken together, this demonstrates that KSHV lytic reactivation stimulates eIF4F assembly by selectively recruiting eIF4G to cap-bound eIF4E. The increased amount of eIF4G bound to eIF4E was not accompanied by detectable changes in the overall steady state levels of eIF4E, eIF4G, eIF4A, PABP, or 4E-BP1 in either input samples or whole cell extracts (Fig. 4A, 4B). However, the basal levels of these factors was much greater when equivalent numbers of live, transformed PEL cells were compared with primary human dermal fibroblasts despite their similar levels of RhoGDI and Actin (Fig. 4C).

Since eIF4G and the translational repressor 4E-BP1 both bind to the same site on eIF4E, it was surprising that the amount of 4E-BP1 retained by eIF4E remained relatively constant despite a significant increase in eIF4G- binding (Fig. 4A). Analysis of the protein fraction prepared from TPA+DOX-induced TREx BCBL1-RTA cells that was not retained on 7-M GTP Sepharose revealed that it was more enriched in 4E-BP1 hyperphosphorylated isoforms than the input material (Fig. 4D). Conversely, 4E-BP1 hyperphosphorylation was not detected in any samples prepared from the parental TREx BCBL1 cells irrespective of whether they had been induced with TPA+DOX. This demonstrates that hyperphosphorylated 4E-BP1 is ejected from eIF4E upon induction of the KSHV productive growth cycle in a naturally infected PEL-derived B-cell line. Our failure to detect a reduction in 4E-BP1 bound to 7-M GTP Sepharose following reactivation-induced eIF4F assembly likely reflects i) the overall abundance of 4E-BP1 combined with the non-linear chemiluminescent detection methodology; and ii) at least 20% of cells within the population that do not undergo lytic reactivation and maintain the latent gene expression program.

Despite an increase in eIF4G binding to eIF4E upon induction of lytic reactivation, a corresponding increase in PABP, an eIF4G-associated protein, was not observed (Fig. 4A). Analysis of PABP subcellular distribution in TREx BCBL1-RTA cells revealed that PABP is predominately cytoplasmic in latently-infected cells. However, TPA+DOX-induced reactivation redistributed PABP exclusively in cells positive for ORF59, a nuclear, viral DNA polymerase processivity factor (Fig. 4E left panel; [27]). Remarkably, PABP appeared concentrated within DAPI-staining nuclei in cells induced to reactivate (Fig. 4E right panel). Thus, while the amount of PABP detected in association with eIF4F remained constant in cells induced to reactivate, it is possible that PABP redistribution markedly reduced its availability in the cytoplasm and its recruitment to eIF4F complexes, thus contributing to the global changes in the protein synthesis profile observed upon reactivation.

### Accumulation of phosphorylated eIF4E in latently-infected PEL cells induced to reactivate

**Figure 5:**
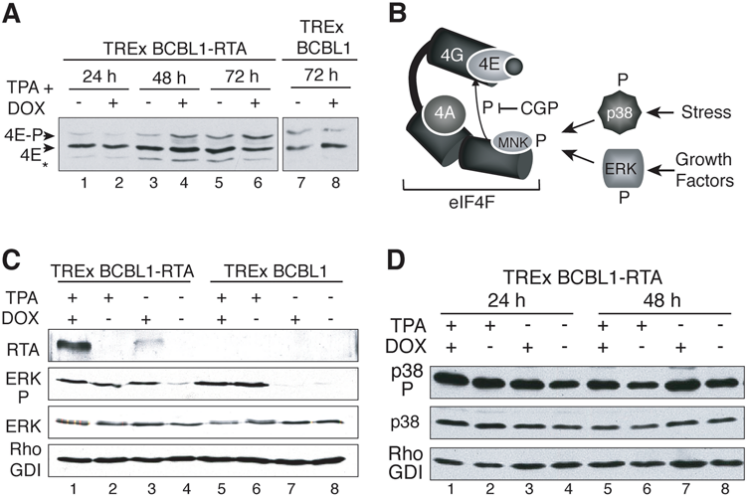
Phosphorylated eIF4E accumulates following KSHV lytic reactivation. A. KSHV reactivation stimulates eIF4E phosphorylation. TREx BCBL1 or TREx BCBL1-RTA cells either untreated (−) or treated (+) with TPA+DOX were collected at the indicated times post-induction. Cytosolic lysates were fractionated by IEF and analyzed by immunoblotting using anti-eIF4E. The arrowheads indicate the different mobilities of the phospho- vs unphosphorylated eIF4E forms [respectively denoted as 4E–P and 4E]. B. Activation of the eIF4G-bound kinase Mnk1 by ERK and p38 in response to diverse stimuli. Active Mnk1 phosphorylates eIF4E when both kinase (Mnk1) and substrate (eIF4E) are bound to eIF4G. C. KSHV lytic reactivation activates ERK. TREx BCBL1 or TREx BCBL1-RTA cells were treated with TPA, DOX or TPA+DOX for 48 h. Total protein was subsequently isolated, fractionated by SDS-PAGE and analyzed by immunoblotting using antibodies specific for RTA or ERK [phospho (denoted as ERK P) *vs.* total ERK]. RhoGDI: loading control. D. p38 is activated in latently-infected cells, and remains active upon KSHV lytic reactivation. Same as in B, except the antisera was specific for phospho- (denoted as p38 P) or total p38.

As a consequence of eIF4F assembly, eIF4G-associated polypeptides, including the eIF4E-kinase Mnk1, are tethered to the 5′ mRNA terminus by eIF4E. To investigate the impact of lytic reactivation on eIF4E phosphorylation, cytosolic lysates from TREx BCBL1-RTA or TREx BCBL1 cells treated with TPA+DOX for various times were fractionated by isoelectric focusing (IEF) to resolve the phosphorylated and unphosphorylated isoforms. The relative abundance of phosphorylated vs unphosphorylated eIF4E was then evaluated by immunoblotting using anti-eIF4E antiserum. Phosphorylated eIF4E accumulates over a 72 h period following the induction of reactivation in TREx BCBL1-RTA cells as the relative ratio of phosphorylated to unphosphorylated eIF4E increases (Fig. 5A) while the total steady-state eIF4E level remains relatively unchanged (Fig. 4B). TPA+DOX treatment of the parental TREx BCBL1 line did not result in a detectable increase in phosphorylated eIF4E. Together, these results establish that reactivation of latent KSHV genomes in PEL cells promotes the accumulation of phosphorylated eIF4E, and suggest that either a virus-encoded or virus-induced gene product plays a critical role in this process.

Phosphorylation of eIF4E involves eIF4F assembly, which brings together the kinase, Mnk1, and its substrate, the cap-binding protein eIF4E, both of which associate with eIF4G [28],[29]. In addition, Mnk1 must be activated by upstream signaling components responsive to multiple biological stimuli or stresses including the mitogen-activated protein kinases (MAPKs) extracellular regulated kinase (ERK) and / or p38 (Fig. 5B) [30]–[32]. To evaluate MAPKs activation during KSHV lytic reactivation, lysates from TREx BCBL1 or TREx BCBL1-RTA cells treated with TPA, DOX or a combination of both, were fractionated by SDS-PAGE and analyzed by immunoblotting. Greater levels of phospho-ERK were observed in TREx BCBL1 and TREx BCBL1-RTA cells after treatment with TPA+DOX or TPA alone (Fig. 5C, lanes 1,2,5 and 6) in agreement with other reports [33],[34]. To distinguish between TPA-mediated and KSHV-mediated ERK activation, TREx BCBL1 and TREx BCBL1-RTA cells were treated with DOX alone prior to evaluating ERK activation. Figure 5C demonstrates that DOX-induced RTA expression in TREx BCBL1-RTA (lane 3) was sufficient to increase phospho- ERK levels compared to untreated cells (lane 4), without a discernible impact on total ERK levels (Fig. 5C, lane 3 and 4). In contrast, DOX-treatment of TREx BCBL1 cells did not activate ERK detectably (Fig. 5C, lanes 7 and 8). Together, these observations strongly suggest that induction of RTA expression in PEL cells latently infected with KSHV triggers ERK activation. Unlike ERK, phosphorylated p38 is detected in latently-infected cells and its levels do not vary significantly after lytic cycle induction (Fig. 5D). Thus, induction of RTA expression and subsequent KSHV reactivation from latency in TREx BCBL1 cells promotes ERK phosphorylation without significantly altering p38 activation. This demonstrates that two kinases required for Mnk1 signaling are active in PEL cells induced to reactivate; however, only ERK activation is directly RTA-responsive and correlates with entry into the viral lytic program.

### Post-transcriptional control of the viral lytic activator RTA by the cellular eIF4E-kinase Mnk1

**Figure 6:**
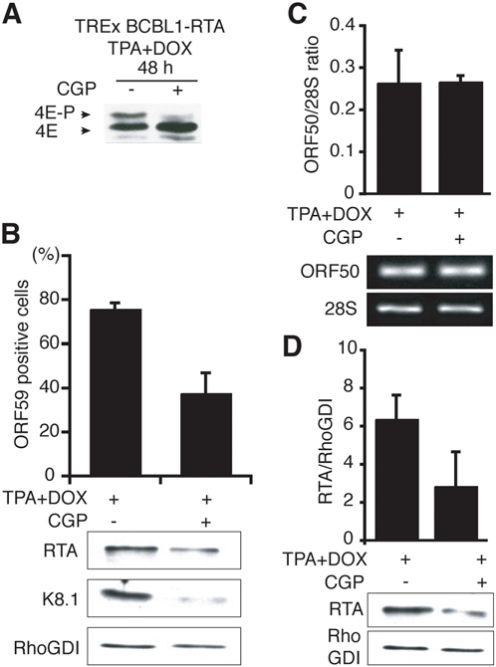
Regulation of RTA abundance by the eIF4E-kinase Mnk1. A. Mnk-inhibitors prevent eIF4E phosphorylation during lytic reactivation of PEL-cells. TREx BCBL1-RTA cells were induced with TPA+DOX in presence (+) or absence (−) of the Mnk inhibitor CGP57380 (20 nM). After 48 h, total protein was isolated, fractionated by IEF and analyzed by immunoblottting as described in the Fig. 5 legend. B. Suppression of KSHV reactivation by Mnk-inhibitors. Top panel: TREx BCBL1-RTA cells were treated as described in A. The fraction of ORF59-positive cells was quantified as described in the legend to Fig. 1. Bottom panel: Lysates from cells treated as in A were fractionated by SDS-PAGE and analyzed by immunoblotting using the indicated antisera. RhoGDI: loading control. C. Treatment with CGP57380 does not affect ORF50 (RTA) mRNA levels. Total RNA was extracted from cells treated as described in panel A and reverse transcription performed on 250 ng of total RNA from each sample. PCR reactions were then performed on 1% of the resulting cDNAs using specific primers for ORF50 (RTA) or 28S rRNA. ORF50 transcripts originating from the viral genomic locus and the DOX-regulated cDNA generate identical amplification products, the latter being the more abundant. Samples were quantified by gel densitometry. Error bars: Standard deviations from independent triplicate experiments. D. Mnk-inhibitors reduce RTA protein accumulation in response to a reactivation signal. Total protein lysates from cells treated as described in C were fractionated by SDS-PAGE and analyzed by immunoblotting using the indicated antisera. Samples were quantified by gel densitometry. Error bars: Standard deviations from independent triplicate experiments.

While not required for normal cell growth and development [35], Mnk1 contributes to proper translational control and is required for wild-type levels of viral replication in acutely infected cells [7],[36],[37]. Involvement of Mnk1 activity in viral reactivation from latency has not been reported. To determine if Mnk1 activity contributes to KSHV reactivation from latency, accumulation of lytic replication markers was measured in TREx BCBL1-RTA cultures induced to reactivate in the presence and absence of the small-molecule Mnk1-inhibitor CGP57380. Total lysates from cells induced in the presence of CGP57380 were fractionated by IEF or SDS-PAGE followed by immunoblotting to evaluate the effectiveness of the Mnk1-inhibitor in this system. Figure 6A clearly shows that phosphorylated eIF4E was not detected in CGP57380-treated cultures under these conditions. Inhibiting Mnk1 activity during KSHV reactivation impaired viral entry into the lytic cycle as assessed by ORF59 immunostaining and significantly reduced the overall abundance of the viral trans-activator RTA along with the late glycoprotein K8.1 (Fig. 6B). Furthermore, the reduced RTA polypeptide abundance in CGP57380-treated cultures was not accompanied by a detectable decrease in the corresponding steady-state mRNA levels as measured by RT-PCR (Fig. 6C,D). These experiments demonstrate that KSHV reactivation is impaired by inhibiting the cellular eIF4E kinase Mnk1, and suggest that Mnk1 regulates the accumulation of RTA, an essential master regulator of KSHV lytic reactivation, via a post-transcriptional mechanism.

## Discussion

Reactivation of latent herpesvirus infections in response to environmental signals requires the abrupt implementation of a new developmental gene expression program. While the onset of this new program typically begins with a burst of new viral gene transcription, proper control of cellular translational regulatory pathways is likely to be critical for its execution. PEL-derived B-cells naturally infected with KSHV provide a valuable model system to study herpesvirus reactivation from latency [38]. However, up until now, it has not been possible to determine how host translational control pathways contribute to or respond to herpesvirus reactivation because of the limited efficiency with which PEL-cells naturally infected with KSHV can be induced to reactivate. By establishing conditions that promote high-level reactivation of latent KSHV from PEL-cells, we demonstrate that reactivation profoundly changes the ongoing protein synthesis program in these cells. Inactivation of the translational repressor 4E-BP1, PABP nuclear accumulation, eIF4F assembly, and phosphorylation of the cap-binding protein eIF4E by the cellular eIF4G-asociated kinase Mnk1 occur in parallel with alterations to the protein synthesis profile. Inhibiting Mnk1 reduces accumulation of the critical viral transactivator RTA through a post-transcriptional regulatory mechanism, limits the production of downstream lytic proteins, and impairs the overall efficiency of viral reactivation.

The latent-lytic replication switch triggers substantial changes in the ongoing protein synthesis profile. PABP redistribution from the cytoplasm to the nucleus in response to lytic reactivation could contribute to this. The KSHV-encoded vIRF4 (K10/10.1) polypeptide expressed during the lytic cycle reportedly associates with PABP and might mediate these effects either alone or in conjunction with other viral factors [39]. A different tactic to rapidly switch the profile of newly synthesized proteins in reactivating cells might involve SOX, the KSHV ORF37 gene product shown to impair protein synthesis in immortalized endothelial cells by accelerating global mRNA turnover [25]. Faster host and viral mRNA-turnover appears to be a common strategy used by different viruses to comprehensively impair mRNA translation [25], [40]–[42] and is capable of accentuating the transition between different temporal classes of viral transcripts [43],[44]. Under these conditions, viral mRNA translation is maintained and appears selective simply because viral gene transcription proceeds at a high level, continually replenishing the mRNA pool compared to the bulk of host mRNAs [45]. Significantly, certain host mRNAs escape this restriction and can be translated, potentially contributing to or antagonizing viral replication [41]. Finally, all herpesvirus family members encode a post-transcriptional regulator essential for lytic replication that represses export of host intron-containing mRNAs, but shuttles between the nucleus and cytoplasm to promote export of viral mRNAs, the vast majority of which lack introns [46]–[49]. Thus, changes to the protein synthesis profile resulting from lytic reactivation are likely achieved through the synergistic action of multiple, independent viral functions.

While reactivation dramatically impairs overall ongoing protein synthesis, we now establish that multiple aspects of the host translation machinery are activated rather than inhibited. From the virus standpoint, this makes sense because the translation apparatus must engage viral mRNAs to execute the lytic developmental program. Whereas modification of translation initiation factors required for cap-dependent mRNA translation occurs in cells acutely infected with related alpha (HSV-1) or beta (HCMV) herpesviruses [7],[26],[37], or poxviruses (vaccinia) [36], this is the first demonstration that reactivation from latency activates the host translation machinery.

Normally, hyperphosphorylation of the 4E-BP1 translational repressor by mTORC1 releases the cap-binding protein eIF4E, allowing eIF4E to associate with eIF4G and assemble the eIF4F complex. Unexpectedly, while KSHV lytic reactivation stimulated the rapamycin-sensitive kinase mTORC1, 4E-BP1 hyperphosphorylation and eIF4F assembly, complete 4E-BP1 ejection from eIF4E was not observed. While the 4E-BP1 fraction released from eIF4E was enriched in hyperphosphorylated isoforms, the remaining eIF4E-bound fraction is likely hyphosphorylated. Despite the relatively high efficiency with which our conditions promote reactivation, lytic gene expression was not detected routinely in at least 20% of the cells. Given the high levels of eIF4E, eIF4G and 4E-BP1 in uninduced TREx BCBL1-RTA cells, the fraction of hypophosphorylated 4E-BP1 retained by eIF4E is likely derived from the sub-population of cells that have not initiated lytic reactivation. Elevated 4E-BP1 levels in cancer cells are not incompatible with ongoing translation, and in fact promote the translation of select mRNAs involved in angiogenesis and cell growth [50]. We cannot, however, exclude that 4E-BP1 inactivation is incomplete in cells induced to reactivate, and this might foster a balance between cap-dependent and cap-independent translation of select host and viral mRNAs. Regardless of which of these alternatives proves correct, we have shown that the repressor 4E-BP1 is stably bound to eIF4E in these latently-infected cells, and viral reactivation results in its partial release with an accompanying eIF4G-recruitment into an eIF4E-containing complex. Furthermore, rapamycin blocked 4E-BP1 phosphorylation without detectably reducing lytic reactivation. eIF4E levels in excess of 4E-BP1 could allow for ongoing cap-dependent translation in these cells. The absence of detectable hyperphosphorylated 4E-BP1 and p70S6K (unpub. obs.) in latently-infected TREx BCBL1 and TREx BCBL1-RTA cell lines raises questions as to the involvement of mTORC1 on their proliferation in culture. Rapamycin reportedly inhibits proliferation of KSHV-positive PEL-lines [51],[52] and the KSHV-encoded vGPCR G-protein coupled receptor is implicated in endothelial cell transformation by inactivating the tuberous-sclerosis complex (TSC), an upstream negative-regulator of mTORC1 [53]. Paradoxically, vGPCR expression in PEL-lines causes G1 arrest, consistent with observations made during the herpesvirus lytic replication cycle, but incompatible with a potential role as an oncogene driving proliferation of latently-infected PEL cells [54]. vGPCR transcripts are expressed as an early lytic mRNA in PEL-cell lines, but low levels are also detected in PEL clinical samples [55]. Whether the signal detected was derived from the truly latent component of the population or a fraction undergoing spontaneous reactivation has not been established. Notwithstanding numerous differences between endothelial and B-cells, our work supports the notion that a virus-encoded function such as vGPCR activates mTORC1 and stimulates 4E-BP1 hyperphosphorylation upon induction of KSHV lytic reactivation. This contrasts with proliferating B-cell lines latently infected with the related γ-herpesvirus EBV, where the latency protein LMP-2 activates PI3-kinase /Akt, resulting in 4E-BP1 hyperphosphorylation by mTORC1 [56].

In addition to inactivating the repressor 4E-BP1 through mTORC1, KSHV reactivation activates ERK 1/2 MAP-kinase and its downstream target Mnk1, the cellular eIF4G-associated, eIF4E-kinase. In cells acutely infected with poxviruses or related herpesvirus family members HSV-1 and HCMV, Mnk1-mediated eIF4E phosphorylation enhances viral protein accumulation, viral replication and spread [7],[36],[37]. In addition, eIF4E overexpression can contribute to malignant transformation [57], and more recently, eIF4E phosphorylation has been implicated in tumorigenesis [58]. Here, we now establish that inhibiting Mnk1 prevents eIF4E phosphorylation and reduces KSHV reactivation from latency. Moreover, we demonstrate that Mnk1 activity post-transcriptionally regulates Rta abundance. Increased phosphorylated eIF4E abundance mediated by Mnk1 activity likely reflects eIF4F complex assembly on the newly transcribed Rta 5′ mRNA terminus, as eIF4E phosphorylation occurs following eIF4F assembly when both the kinase Mnk1 and its substrate, eIF4E, are bound to eIF4G [26]. While Mnk1 and eIF4E phosphorylation are not essential for translation, they might impart a level of control that governs the efficiency with which translation initiates on newly minted or derepressed mRNAs. Should this hypothesis prove true, contributions by Mnk1 and / or eIF4E phosphorylation to the efficiency with which newly transcribed or derepressed mRNAs are translated is likely not to be confined to viral biology, but may also be incorporated into a repertoire of translation control strategies important in health, stress responses, and disease.

## Materials and Methods

### Cells and antibodies

TREx BCBL1 and TREx BCBL1-RTA (kindly provided by Dr. Jae Jung, USC, Los Angeles, CA) were maintained in DMEM supplemented with 20% Fetalplex (Gemini), L-glutamine (200 µM, Invitrogen), penicillin (100 U/ml, Gibco) and streptomycin (100 µg/ml, Gibco). TRExBCBL1-RTA cells were grown in presence of hygromycin B (100 µg/ml) to maintain selection for the ORF50 (RTA) gene.

The following antibodies were purchased from commercial suppliers: Cell Signaling Technologies [anti-p38 (Cat. 9212), phospho-p38 (Cat. 9216S), p41/p42 ERK (Cat. 9102), phospho-ERK (Cat. 9101S)]; Bethyl Labs [anti-4EBP1 (Cat. A300-501A)]; BD Transduction labs [anti-eIF4E (Cat. 610269)]; Santa Cruz Biotechnology [anti-RhoGDI (Cat. SC-360)]; Adv. Biotechnologies Inc. [anti-KSHV K8.1 (Cat. 13-212-100)]. The following antibodies were generous gifts: anti-ORF59, Dr. Bala Chandran (Rosalind Franklin Univ., North Chicago, Ill); anti-eIF4A mouse monoclonal, Dr. Robert Schneider (NYU School of Medicine, New York, NY); anti-RTA, Dr. Gary Hayward (Johns Hopkins School of Medicine, Baltimore, MD).

### Induction of KSHV lytic replication in PEL-derived cells

TREx BCBL1 and TREx BCBL1-RTA cells were seeded at 1.5×10^5^ cells/ml. 24 h after seeding, cells were induced with 2-*O*-tetradecanoylphorbol-13-acetate (TPA 20 ng/ml, Cat. P1585, Sigma), doxycycline (2 µg/ml, Cat. 631311, BD Bioscience) or both TPA+DOX. Where indicated, cells were pretreated for 1 h with CGP57380 (20 µM, Cat. 454861 Calbiochem) or rapamycin (100 nM, Cat. 553210 Calbiochem), followed by induction in presence of these drugs. At the indicated times after induction, 3×10^5^ live cells were collected, resuspended in 125 µl of SDS lysis buffer (62.5 mM Tris-HCl, pH 6.8, 2% SDS, 10% glycerol, 0.7 M β–mercaptoethanol) and boiled for 3 min.

### Immunofluorescence analysis

TREx BCBL1 or TREx BCBL1-RTA cells were attached to poly-L-lysine-treated (0.1% for 1 h) coverslips. Cells were fixed in 3.7% formaldehyde, permeabilized with 0.1% Triton-X100+RNase A (100 µg/ml), blocked with PBS-B [PBS+10% FBS+0.25% saponin], and subsequently incubated with primary antibody diluted in PBS-B for 1 h at 37°C. Cells were washed and probed with secondary antibody diluted in PBS-B for 1 h at 37°C. DNA was stained with propidium iodide or DAPI. Coverslips were mounted onto glass slides with Dako, and fluorescent images collected with a Carl Zeiss LSM510 Meta confocal laser-scanning microscope or a Zeiss Axiovert fluorescence microscope, using metamorph software.

### Analysis of protein synthesis in TREx BCBL1 and TREx BCBL1-RTA cells

At the indicated times post-induction, 6×10^5^ live TREx BCBL1 or TREx BCBL1-RTA cells were incubated for 30 min with 7 mCi/ml of *^35^S Express* (Perkin Elmer) in serum-free DMEM lacking methionine+cysteine. Cells were collected, lysed in 250 µl of SDS lysis buffer and the samples boiled for 3 min. An aliquot of the cell lysate was fractionated by SDS-PAGE in a 12.5% polyacrylamide gel. The gel was fixed, dried and exposed to X-OMAT X-ray film overnight.

### Immunoblotting , isoelectric focusing and 7-Methyl GTP chromatography

Immunoblotting and isoelectric focusing (IEF) were performed as previously described [7]. Isolation of eIF4E-bound proteins by 7-Methyl GTP batch chromatography was essentially performed as previously reported [37] in lysates from 1.5×10^6^ live TREx-BCBL-1 or TREx-BCBL-1/RTA cells 48 h post-induction with TPA+DOX.

### Semi-quantitative and real-time RT-PCR

Total RNA was extracted from 1×10^6^ TREx BCBL1-RTA cells induced for 48 h with TPA+DOX in the presence or absence of 20 nM CGP57380 using Trizol reagent (Invitrogen). Reverse transcription was performed on 250 ng of each RNA with SuperScript First-Strand Synthesis System (Invitrogen) and 1% of the cDNA product was used for PCR amplification. Products were separated on a 2% agarose gel and quantified by scanning densitometry. Spliced ORF50 (RTA) transcripts were detected using primers [59] located in the 5′ UTR (5′-CACAAAAATGGCGCAAGATGA-3′) and second exon (5′-TGGTAGAGTTGGGCCTTCAGTT-3′). These primers do not distinguish between transcripts derived from the viral genomic locus and the Dox-regulated cDNA present in TREx BCBL1-Rta cells. 28S RNA served as a normalization control and was amplified using the following primers: 5′-AAACTCTGGTGGAGGTCCGT-3′, 5′-CTTACCAAAAGTGGCCCACTA-3′.

Changes in the abundance of representative cellular mRNAs in DMSO-treated or TPA+DOX-induced cells was measured by real-time RT-PCR analysis using a Lightcycler 2.0 (Roche Applied Science) and the following primer pairs: β-actin 5′-GCGGGAAATCGTCGGTGACATT-3′, 5′-GATGGAGTTGAAGGTAGTTTCGTG-3′; glyceraldehyde 3-phosphate dehydrogenase (GAPDH) 5′-CCTCAACGACCACTTTGTCA-3′, 5′-CCCTGTTGCTGTAGCCAAAT-3′; interleukin-2 (IL-2) 5′-CCCAGGGACTTAATCAGCAA-3′, 5′-GGTTGCTGTCTCATCAGCAT-3′; and β-catenin 5′-CCCACTGGCCTCTGATAAAGG-3′, 5′-ACGCAAAGGTGCATGATTTG-3′. Samples were normalized using 28S RNA.

## Supporting Information

Figure S1 TPA+DOX treatment of TREx BCBL1-RTA cells results in the production of infectious KSHV particles. A. Using a luciferase reporter cell line to detect infectious KSHV produced by TREx BCBL1-RTA cultures in the presence and absence of inducer. Asynchronous cultures of TREx BCBL1-RTA cells (150 ml total seeded at 2×10^5^ cells /ml) were induced with TPA+DOX or mock-induced using DMSO as described. After 6 days, a cell-free lysate was prepared by freeze-thawing and cell debris was removed by centrifugation (800×g for 10 min at 4°C). After passage through a 0.8 µ filter, the supernatant was floated on top of a 7 ml cushion of 50 mM Tris-HCl, pH 7.2, 1 mM MgCl_2_, 20% sorbitol and centrifuged at 20,000 rpm for 90 min in a SW28 rotor at room temperature. The viral pellet was resuspended in 0.5 ml DMEM+1.5% fraction V BSA and 0.125 ml was applied to approximately 1.5–3×10^5^ 293 PAN-luc reporter cells. After 48 h, the cells were harvested and the luciferase activity present in a fraction of the sample was measured (shown in B) using commercially available reagents (Promega).
(1.01 MB TIF)
