# Supplementary figures and images for: Activation of Host Translational Control Pathways by a Viral Developmental Switch
Source: PLoS Pathog. 2009 Mar 20;5(3):e1000334. doi: 10.1371/journal.ppat.1000334 (PMC2652079; doi:10.1371/journal.ppat.1000334)

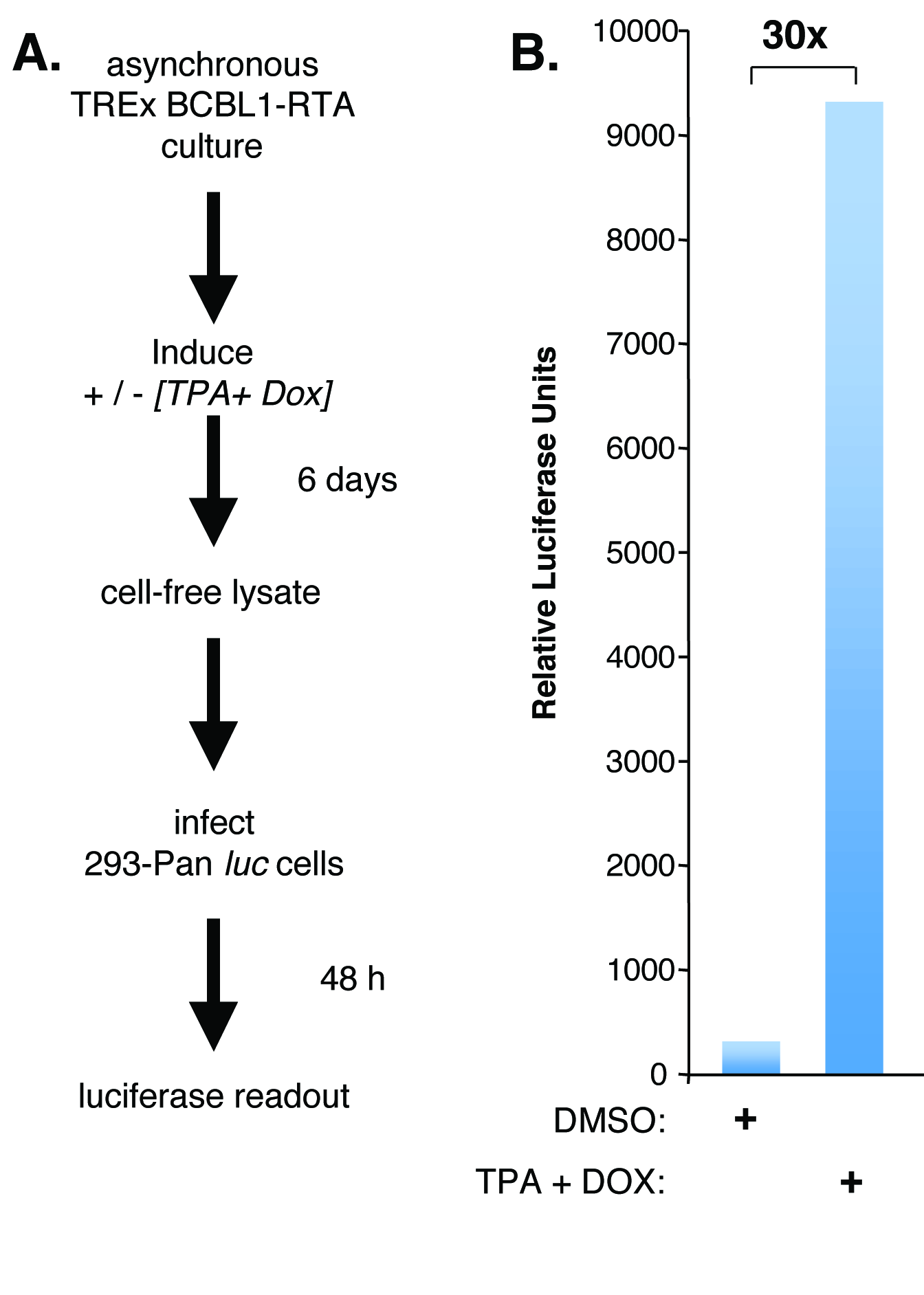

Supplement: Figure S1 — TPA+DOX treatment of TREx BCBL1-RTA cells results in the production of infectious KSHV particles. A. Using a luciferase reporter cell line to detect infectious KSHV produced by TREx BCBL1-RTA cultures in the presence and absence of inducer. Asynchronous cultures of TREx BCBL1-RTA cells (150 ml total seeded at 2×10^5^ cells /ml) were induced with TPA+DOX or mock-induced using DMSO as described. After 6 days, a cell-free lysate was prepared by freeze-thawing and cell debris was removed by centrifugation (800×g for 10 min at 4°C). After passage through a 0.8 µ filter, the supernatant was floated on top of a 7 ml cushion of 50 mM Tris-HCl, pH 7.2, 1 mM MgCl2, 20% sorbitol and centrifuged at 20,000 rpm for 90 min in a SW28 rotor at room temperature. The viral pellet was resuspended in 0.5 ml DMEM+1.5% fraction V BSA and 0.125 ml was applied to approximately 1.5–3×10^5^ 293 PAN-luc reporter cells. After 48 h, the cells were harvested and the luciferase activity present in a fraction of the sample was measured (shown in B) using commercially available reagents (Promega). (1.01 MB TIF) [file ppat.1000334.s001.tif]
